# Association between suicidal behaviour and impaired glucose metabolism in depressive disorders

**DOI:** 10.1186/s12888-015-0567-x

**Published:** 2015-07-22

**Authors:** Hannu Koponen, Hannu Kautiainen, Esa Leppänen, Pekka Mäntyselkä, Mauno Vanhala

**Affiliations:** Old Age Psychiatry, University of Helsinki, and Helsinki University Hospital, P.O. Box 22, FIN-00014 Helsinki, Finland; Primary Health Care Unit, Kuopio University Hospital, Kuopio, Finland; Primary Health Care Unit, Central Hospital of Central Finland, Jyväskylä, Finland; Public Utility Laboratory KESLAB, Central Finland Hospital District, Jyväskylä, Finland; Primary Health Care Unit, Institute of Public Health and Clinical Nutrition, School of Medicine, University of Eastern Finland, Kuopio, Finland; Department of Health Sciences, University of Eastern Finland, Kuopio, Finland

**Keywords:** Cholesterol, Depression, Glucose, Insulin resistance, Suicidal behaviour, Ideation, Suicidality, Suicide attempt, Triglyceride

## Abstract

**Background:**

Disturbances in lipid metabolism have been linked to suicidal behaviour, but little is known about the association between suicide risk and abnormal glucose metabolism in depression. Hyperglycaemia and hyperinsulinaemia may increase the risk of depression and also the risk for suicide, we therefore studied associations between suicidal behaviour and disturbances in glucose metabolism in depressive patients who had been referred to depression nurse case managers.

**Methods:**

Patients aged 35 years and older (*N* = 448, mean age 51 years) who were experiencing a new depressive episode, who were referred to depression nurse case managers in 2008–2009 and who scored ≥10 on the Beck Depression Inventory were enrolled in this study. The study was conducted in municipalities within the Central Finland Hospital District (catchment area of 274 000 inhabitants) as part of the Finnish Depression and Metabolic Syndrome in Adults study. The patients’ psychiatric diagnoses and suicidal behaviour were confirmed by the Mini-International Neuropsychiatric Interview. Blood samples, for glucose and lipid determinations, were drawn from participants after 12 h of fasting, which was followed by a 2-hour oral glucose tolerance test (OGTT) when blood was drawn at 0 and 2 h. Insulin resistance was measured by the Quantitative Insulin Sensitivity Check Index (QUICKI) method.

**Results:**

Suicidal ideation (49 %) and previous suicide attempts (16 %) were common in patients with major depressive disorder or dysthymia. Patients with depression and suicidal behaviour had higher blood glucose concentrations at baseline and at 2 hours in the OGTT. Glucose levels associated positively with the prevalence of suicidal behaviour, and the linearity was significant at baseline (p for linearity: 0.012, adjusted for age and sex) and for 2-hour OGTT glucose (p for linearity: 0.004, adjusted for age and sex). QUICKI levels associated with suicidal behavior (p for linearity across tertiles of QUICKI: 0.026). Total and LDL cholesterol and triglyceride levels were also higher in those patients with suicidal behaviour. Multivariate analysis revealed that blood glucose levels, BDI scores and antidepressive medications associated with suicidal behaviour.

**Conclusion:**

Insulin resistance and disturbances in glucose and lipid metabolism may be more common in middle-aged depressive patients with suicidal behaviour.

## Background

Depression is thought to have multifaceted pathophysiology and increased risk for having depression is related to type two diabetes and other cardiometabolic disorders [1–3]. Numerous studies have also documented an association between suicide risk and depression [4, 5]. Increased suicide risk in depression may be related to various psychosocial factors, interpersonal conflicts and negative life events [6]. However, recent studies have reported an association between diabetes, depressive disorders and suicidal ideation [7, 8], in addition to an association between higher high glycated haemoglobin (HbA1c) values and suicidal ideation in subjects with diabetes [9]. Another cardiometabolic risk factor, disturbances in lipid metabolism, may also associate with impulsiveness and suicidal behaviour [10].

The monoamine hypothesis states that depression is related to monoaminergic dysfunction [11]. Reduced serotonin levels associate with increased weight, increased waist circumference, elevated blood glucose levels and insulin resistance in addition to depressed mood [12]. The inflammatory hypothesis of depression emphasizes the role of pro-inflammatory cytokines and cell-mediated immune activation that results in tryptophan depletion and serotonergic hypofunction [13–16]. Type 2 diabetes is recognized as an immune-mediated disease in which cytokines play an important role that leads to impaired insulin signaling and to the selective destruction of the insulin-producing beta cells [17]. A possible underlying pathophysiological mechanism for the emergence of abnormal glucose metabolism in depression and suicidal behaviour could thus lie in the interplay between serotonin and proinflammatory cytokines [18–20].

Studies on the associations between depression, suicidal behaviour, glucose levels or insulin resistance are scarce, although some previous studies suggest that higher glucose levels are associated with dysthymia [7] and higher HbA1c concentrations with recurrent or psychotic depression [7]. Bot and co-workers reported an association between suicidal ideation and HbA1c levels in diabetic subjects [9]. A recent meta-analysis reported an association between depression and insulin resistance [21]. The role of insulin secretion is more obscure, though a longitudinal study found that low insulin secretion is a risk factor for depression in middle-aged women [22]. Hyperglycaemia and insulin resistance may increase the risk of depression and also the risk of suicide, thus, we decided to study the associations between suicidal behaviour and disturbances in glucose metabolism in depressive patients that had been referred to depression nurse case managers.

## Methods

Patients who were 35 years of age or older, who were having a new depressive episode, who were referred to depression nurse case managers in 2008–2009 and who also scored ≥10 on the Beck Depression Inventory (BDI) [23] were enrolled in this study. The study (Finnish Depression and Metabolic Syndrome in Adults (FDMSA) study) was conducted in both urban and rural municipalities that were part of the Central Finland Hospital District, Finland with a geographically unified catchment area of 274 000 inhabitants. Enrolment was based on written and oral patient information. Each enrolled patient gave her/his written consent. The Ethics Committee of Central Finland Hospital District had approved the study protocol prior to the commencement of the study. Depression and type two diabetes are more prevalent in the Finnish population after 35 years of age, therefore we decided to focus on the ≥35 year-old age group. The age limit of 35 years was also chosen to obtain a stable study population, which facilitates having a more prolonged follow-up. We obtained a representative sample comprising both mild and more severely depressive participants, by including self-referred patients along with general practitioner referred patients. In Finland, the depression nurse case managers are an important part of the basic health care team and they primarily organize the treatment of depressed patients together with the general practitioners. The patients were treated according to the current Finnish treatment guidelines for depression, i.e., with antidepressive medications and/or psychotherapy. If suicidal behaviour was a serious current problem, then those suicidal patients were further referred to a psychiatric hospital.

All participants completed a standard questionnaire that contained questions about their use of medications, which *inter alia* included antidepressant use and also hormone replacement therapy in females. In addition, data were collected on smoking habits, alcohol consumption (number of drinks per week) and physical activity (number of over-30-min exercise sessions per week). The severity of depressive symptoms was ascertained by scoring the responses to the 21-item BDI [23], which all patients had to complete and the psychiatric diagnosis was subsequently confirmed upon having a diagnostic interview. The diagnostic interview was the Mini-International Neuropsychiatric Interview (M.I.N.I.) [24]. The M.I.N.I. is a structured diagnostic interview that is compatible with the International Statistical Classification of Diseases and Related Health Problems, Tenth Revision (ICD-10), and the Diagnostic and Statistical Manual of Mental Disorders, Fourth Edition (DSM-IV). Suicidal behaviour was defined in the present study according to the M.I.N.I. suicidal behaviour module as ‘suicidal ideation during the past month’ or ‘suicide attempt during the past month or in a lifetime’. A total of 706 referred patients scored 10 or more for the BDI. However, only 448 patients were selected for the study population after having the diagnostic interview after which they were deemed to have met the criteria for major depressive disorder or for dysthymia.

Fasting blood samples were drawn between 8 and 11 o’clock after 12 h of fasting for glucose and lipid level determinations. This was followed by a 2-hour oral glucose tolerance test at 0 and 2 h sampling (OGTT). The physical examination included the participants’ weight, height, waist circumference and blood pressure measurements, which were taken during the same study visit. Height and weight of the participants were determined with the participants wearing light clothing and was accurate to the nearest 0.5 cm and 0.1 kg, respectively. The waist circumference was measured to the nearest 1.0 cm at the midpoint between the lateral iliac crest and the lowest rib. Trained nurses measured blood pressure twice with a mercury sphygmomanometer while patients/participant who had first been rested for 15 min were in a sitting position.

Modular Analytics SWA (Hitachi High-Technologies Corporation, Tokyo, Japan) served to determine serum total cholesterol, HDL cholesterol, LDL cholesterol, triglycerides and plasma glucose. The concentrations of serum insulin were analysed by a Siemens Advia Centaur (Siemens Health Care Diagnostics, Dublin, Ireland). We applied all the routine quality control procedures of an accredited clinical laboratory (Central Finland Hospital District, Clinical laboratory) including regular testing of standard samples. The sensitivity of the insulin assay was 2.4 U/l, and the intra- and inter-assay coefficients of variation were 5.3 % and 7.6 %, respectively. Insulin resistance may co-occur with depression, therefore we also calculated the Quantitative Insulin Sensitivity Check Index (QUICKI) on the basis of the insulin and glucose levels according to the equation: QUICKI = 1/[log(I0) + log (G0)], in which I0 is fasting insulin, and G0 is fasting glucose [25]. In QUICKI, low values are associated with insulin resistance.

### Statistical analysis

The results are presented as means, interquartile ranges, standard deviations and frequency distributions. We used the Student’s *t*-test, Mann–Whitney-*U*-test and the chi-square test to test for statistical significance between groups. The multivariate logistic regression model was used to investigate factors related to suicidal behaviour, and clinically relevant variables without covariability were selected from Table 1 for the procedure. We then used generalized linear models with the appropriate distribution and link functions to evaluate the statistical significance for the hypotheses of linearity. Any violations of the assumptions (non-normality), were evaluated by a bootstrap-type test. The normality of the variables was tested by using the Shapiro-Wilk W test.

**Table 1:** Groups taking part in the FDMSA study

	No suicidal ideation and behaviour *N* = 230	Suicidal ideation and attempts in M.I.N.I. *N* = 218	*p*
Women, n (%)	169 (74)	143 (66)	0.059
Age, mean (SD)	51 (10)	51 (10)	0.64
Age 65+ years old, n (%)	22 (10)	22 (10)	0.66
Education years, mean (SD)	11.2 (2.9)	11.2 (2.8)	0.95
Working status, n (%)			0.15
Employed	108 (47)	81 (37)	
Retired	61 (27)	62 (28)	
Unemployed	56 (24)	69 (32)	
Student	4 (2)	6 (3)	
Household income, 1000€, median (IQR)	24 (12, 40)	20 (10, 35)	0.50
BMI, mean (SD)	27.9 (5.8)	28.2 (5.9)	0.49
Blood pressure (mmHg), mean (SD)			
Systole	130 (16)	131 (15)	0.45
Diastole	82 (11)	82 (11)	0.90
Plasma Glucose, mmol/l, mean (SD)			
0 h	5.71 (1.17)	6.07 (1.68)	0.013
2 h	5.85 (1.83)	6.69 (3.01)	0.0013
Insu0, U/l, mean (SD)	9.65 (8.08)	10.97 (8.38)	0.11
Insu1, U/l, mean (SD)	58.5 (58.9)	69.3 (69.9)	0.12
QUICKI, mean (SD)	0.16 (0.01)	0.15 (0.01)	0.023
Total cholesterol (mmol/l), mean (SD)	4.97 (0,98)	5.22 (1.06)	0.017
HDL cholesterol (mmol/l), mean (SD)	1.61 (0.50)	1.53 (0.45)	0.089
LDL cholesterol (mmol/l), mean (SD)	2.90 (0.83)	3.21 (1.00)	<0.001
Triglyceride (mmol/l), mean (SD)	1.29 (0.77)	1.48 (0.92)	0.025
BDI score, mean (SD)	21 (7)	26 (8)	<0.001
median (IQR)	20 (15, 25)	26 (20, 32)	
Smoking, n (%)	56 (24)	86 (39)	<0.001
Coronary heart disease, n (%)	12 (5)	9 (4)	0.58
Arterial hypertension, n (%)	69 (30)	64 (29)	0.86
Previous stroke, n (%)	7 (3)	0 (0)	0.015
T2D, n (%)	30 (13)	24 (11)	0.50
Alcohol use disorder, n (%)	21 (9)	39 (18)	0.007
Antidepressive medication, n (%)	139 (61)	170 (78)	<0.001
Statin medication, n (%)	57 (25)	39 (18)	0.007
Leisure time physical activity, n (%)			0.824
Low	79 (35)	71 (33)	
Moderate	56 (25)	50 (24)	
High	91 (40)	92 (43)	

*T2D* type 2 diabetes, *BMI* body mass index, *QUICKI* quantitative insulin sensitivity check index, *Insu0* baseline serum insulin level, *Insu1* serum insulin level after 2-hour OGTT, *BDI* beck depression inventory, *IQR* interquartile range

## Results

Nearly half 218 (49 %) of the 448 participants had suicidal thoughts, whereas 72 (16 %) of participants also had previously attempted suicide according to the results of the M.I.N.I. interview. Participants with depression and suicidal behaviour had higher blood glucose levels at baseline and at 2 hours with the OGTT (Table 1).

**Fig. 1:**
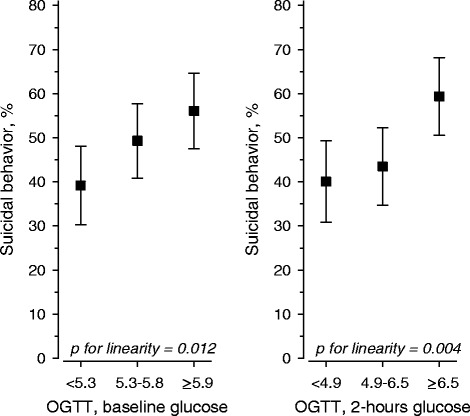
Age and sex-adjusted association between baseline and 2-hour OGTT glucose tertiles and suicidal behaviour

**Fig. 2:**
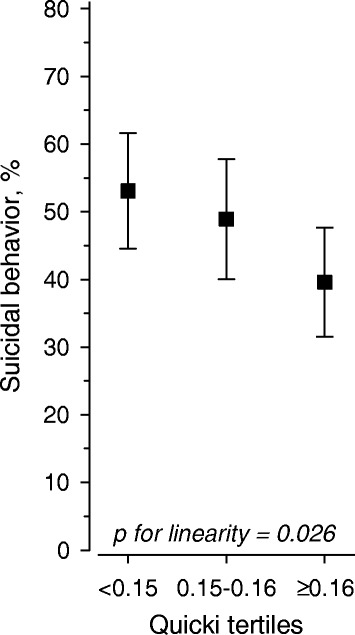
Age and sex-adjusted association between QUICKI tertiles and suicidal behavior

The blood glucose concentrations associated positively with the prevalence of suicidal behaviour (Fig. 1), and linearity was significant for baseline glucose concentrations (p for linearity: 0.012, adjusted for age and sex) and for 2-hour OGTT glucose concentrations (p for linearity: 0.004, adjusted for age and sex adjusted). The OR for suicidal behaviour for the highest vs. the lowest tertile of fasting glucose concentrations at baseline for the OGTT was 2.18 (95 % CI: 1.27–3.74) whereas the corresponding tertile values for 2-hour glucose, OR was 1.99 (95 % CI: 1.64–3.38). The baseline or 2-hour insulin levels were not significantly different from each other, but QUICKI levels showed a significant association with suicidal behaviour (p for linearity across tertiles of QUICKI: 0.026; Fig. 2). When the 72 patients who had a previous suicide attempt were scrutinized separately from the 218 patients who had suicidal ideation, no statistically significant linearity in the OGTT values was observed.

Of the other cardiometabolic risk factors, total and LDL cholesterol and triglyceride levels were higher among participants who had either suicidal ideation or who had previously attempted suicide (Table 1). Patients with suicidal behaviopur also smoked more frequently, and alcohol dependence was also more common (Table 1). In the multivariate analysis, plasma glucose levels, BDI score and antidepressive medication associated with suicidal behaviour. No associations with suicidal behaviour and age, prevalence of type 2 diabetes, gender distribution, smoking, alcohol use disorder, or chronic diseases were found (Table 2).

**Table 2:** Associations between suicidal behaviour and patient characteristics

	OR (95 % CI)	*P*-value
Men	1.58 (0.91 to 2.76)	0.10
Age	0.99 (0.95 to 1.02)	0.36
Education years	1.03 (0.95 to 1.12)	0.44
Working status		
Employed	1 (Reference)	
Retired	2.07 (1.04 to 4.11)	0.038
Un-employed	1.22 (0.69 to 2.16)	0.49
Student	1.34 (0.25 to 7.14)	0.74
BMI	1.02 (0.97 to 1.06)	0.49
Plasma Glucose* tertiles		
I (≤5.2)	1 (Reference)	0.008 (linearity)
II (5.3 to 5.8)	1.33 (0.74 to 2.41)	
III (≥5.9)	2.50 (1.26 to 4.94)	
BDI score	1.10 (1.06 to 1.13)	<0.001
Smoking	1.66 (0.99 to 2.76)	0.051
Coronary heart disease	1.32 (0.43 to 4.10)	0.63
Arterial hypertension	0.84 (0.45 to 1.55)	0.57
T2D	0.69 (0.31 to 1.57)	0.38
Alcohol use disorder	0.81 (0.39 to 1.68)	0.57
Antidepressive medication	2.15 (1.30 to 3.54)	0.003
Statin medication	0.55 (0.29 to 1.05)	0.071
Leisure time physical activity		
Low	1 (Reference)	0.45 (linearity)
Moderate	0.77 (0.43 to 1.39)	
High	0.81 (0.48 to 1.37)	

## Discussion

A novel finding in our study was the observed association between higher plasma glucose/blood glucose levels, insulin resistance and suicidal behaviour in depression. Nearly half of the participants in our study reported suicidal ideation or previous attempts at suicide, which were as frequent as those reported in another recent Finnish study, the Vantaa Depression Study [26]. Cases with a history of suicidal behaviour also had higher triglyceride concentrations in addition to elevated total and LDL cholesterol levels. Statins may slightly increase the risk for developing diabetes [27], but as the use of statins in our study was more prevalent in the subgroup with no suicidal behaviour our finding cannot be therefore attributed to statin medication. The multivariate analysis revealed that plasma glucose levels, BDI scores and antidepressive medications positively associated with suicidal behaviour. No associations with suicidal behaviour and age, prevalence of type 2 diabetes, gender distribution, smoking, alcohol use disorder, or chronic diseases were found however. With the exception of hypertension, cardiovascular diseases (CVDs) were rare due to the comparatively low age of the study population. Although a recent study found an association between obesity and suicidal behaviour [28], our study population showed no differences in body mass index levels.

We observed higher blood glucose for the OGTT both at baseline and at 2-hour sampling in patients with suicidal ideation or who had previously attempted suicide. Ceretta et al. [7] reported three-fold higher suicidal ideation rates in insulin-treated type 2 diabetic patients, which suggested an association between suicidal tendencies and blood glucose levels. Type 2 diabetes was more common in the subgroup with no suicidal behaviour in our study and this suggests that the observed association cannot solely be due to type 2 diabetes. Bot and co-workers [9] also reported an association between higher HbA1c values and depressive mood, sleep disturbance, appetite problems and suicidal ideation. Their study population comprised participants with either type 1 or type 2 diabetes, and the associations were more profound in type 1 diabetes [9]. Han and co-workers have also reported an increased risk for suicidal ideation in adults with diabetes and depression [8], but none of the previous studies have specifically studied the association between glucose levels and suicidal tendencies behaviour in depressed patients.

The association observed between glucose levels and suicidal behaviour may be related to the cytokine-mediated inflammatory process that results in the activation of indoleamine 2,3-dioxygenase, the depletion of tryptophan and suicidal behaviour related to emerging serotonergic hypofunction, depression and impulsivity [13, 17, 29, 30]. The groups in the present study showed no differences in baseline and 2-hour insulin levels, which suggests insulin resistance rather than insufficient insulin secretion was the probable associated factor. In addition, the surrogate index for insulin resistance, QUICKI, showed a significant association with suicidal behaviour, which lends further support for the association between disturbed glucose metabolism and suicidal behaviour. However, the patients with suicidal behaviour in our study also had higher BDI scores, so the severity of depression and the higher prevalence of alcohol use disorder in this group of patients may also have contributed to suicidal behaviour [31, 32]. In addition, hyperactivation of the hypothalamic-pituitary-adrenal axis has been reported in 20 to 80 % of depressed individuals [33]. This hyperactivity has been put forward as an important mechanism that explains both the pathophysiology of depression and also its relationship with medical conditions such as diabetes and obesity. Higher cortisol levels are associated with age and more severe forms of depression, such as melancholic and psychotic depression, and may thus also contribute to the association between higher BDI levels and risk of suicidal behavior [33, 34].

Previous studies have identified smoking as a possible risk factor for attempted and also completed suicide [35]. Smoking has been linked to serotonergic dysfunction [3]. Serum cholesterol has been suggested to reflect the serotonergic function of the central nervous system and to participate in cognitive functioning in addition to mood regulation [10]. Low cholesterol levels have been linked by some studies to impulsiveness and the risk of suicide in depressive patients [10, 36, 37]. However, another study has reported that high cholesterol [38] concentrations to be associated with suicidal behaviour. In this study, we found higher total and higher LDL cholesterol levels in participants with suicidal behaviour, which is in line with the results of Silic et al. [18], who also reported an association between low platelet serotonin, and high interleukin IL-6, and glucose. The higher triglyceride levels were probably related to the insulin resistance or unhealthy lifestyles that were more common in the subgroup with suicidal behaviour [29].

The strengths of our study include its extensive and geographically representative sample of middle-aged and elderly subjects. We also used a diagnostic interview to complement the self-rating of the depressive symptoms. However, we cannot draw inferences about the causality due to the cross-sectional design of our study, which is a limitation. In addition, only persons aged 35 or older were enrolled in the study, so the results cannot be generalized to younger age groups. The evaluation of insulin resistance by QUICKI relying on the mathematical modelling of fasting plasma glucose and insulin concentration may also be a limitation, although a recent study reported that QUICKI results were comparable to a euglycemic hyperinsulinemic clamp and superior to measurements based on insulin alone [39].

## Conclusions

The observed association between plasma glucose levels, insulin resistance and suicidal behaviour suggests that disturbances in glucose metabolism are associated with suicidal ideation and attempts. However, further studies are necessary to elucidate the pathophysiology behind these associations, and it remains obscure whether the treatment of cardiometabolic risk factors lowers the risk of suicidal behaviour.
